# Visual improvement following glaucoma surgery: a case report

**DOI:** 10.1186/1471-2415-14-162

**Published:** 2014-12-23

**Authors:** William S Foulsham, Lanxing Fu, Andrew J Tatham

**Affiliations:** Forth Valley Royal Hospital, Larbert, UK; Princess Alexandra Eye Pavilion and Department of Ophthalmology, University of Edinburgh, Edinburgh, UK

**Keywords:** Glaucoma, Trabeculectomy, Neuroregeneration

## Abstract

**Background:**

Glaucoma is a progressive optic neuropathy and a leading cause of blindness. Neural losses from glaucoma are irreversible, and so the aim of glaucoma treatment is to slow progression and minimize the risk of further damage. Functional improvement with treatment is not expected. We report the case of a patient who experienced a significant improvement in vision following glaucoma surgery and review the literature regarding this phenomenon.

**Case presentation:**

A 64-year old male presented with a 13-month history of gradual vision loss in the right eye to the extent that he could only perceive hand movements. His intraocular pressure (IOP) measured 50 mmHg and he was found to have advanced primary open angle glaucoma. Medical treatment was commenced and he underwent a successful right Mitomycin C-augmented trabeculectomy. Unexpectedly he experienced marked improvement in vision post-operatively, with improvements maintained through six months of follow-up. At his most recent visit visual acuity was 6/18 in the affected eye. Although the mechanism of improved vision cannot be proven it is likely that successful lowering of IOP resulted in some reversal of retinal ganglion cell dysfunction. Important factors may have included his relatively young age, high IOP and short duration of symptoms.

**Conclusion:**

Although rare, functional improvements may occur following trabeculectomy. Glaucoma surgery should be offered early to those with advanced disease, and considered even in those with reduced visual acuity.

## Background

Glaucoma is characterized by progressive optic nerve degeneration, evident clinically as structural changes to the optic nerve head and corresponding loss of visual field [1]. The pathogenesis is not fully understood but it is thought to involve a heterogeneous group of pathological processes that share the final common pathway of progressive death of retinal ganglion cells and their axons [2]. Glaucomatous damage is deemed irreversible; therefore the optimal management currently depends on early detection and treatment to minimize the risk of progression and development of visual loss.

The first line treatment for glaucoma has traditionally consisted of medical management with topical intraocular pressure lowering agents. However, recent guidance, such as that from the United Kingdom National Institute of Clinical Excellence (NICE) stipulate that, for patients with advanced disease at presentation, surgery may be an appropriate first line therapy [3]. The aim of early surgery is to reduce the risk of further deterioration in visual function, as improvement in vision following glaucoma surgery is not expected. There is however, some evidence that retinal ganglion cells damaged by glaucoma might undergo a period of reversible dysfunction preceding cell death [4, 5]. Furthermore, reversible changes in optic nerve head morphology have been reported following reductions of intraocular pressure [6–9]. These observations suggest that certain structural and functional improvements may in fact be possible in some patients.

The aim of the current article is to 1) report the case of a patient with advanced glaucoma at initial presentation that demonstrated marked improvement in visual acuity following reduction in intraocular pressure with trabeculectomy, and 2) to critically appraise the published literature as to whether functional improvement is possible following glaucoma surgery.

## Case presentation

A 64-year-old university professor presented to our facility complaining of a 13-month history of progressive visual loss in his right eye. There was no associated headache or ocular pain and he had no other symptoms of note. He had no previous history of ocular problems, although he had not attended for an eye examination for more than ten years. The patient’s past medical history was significant for bladder cancer managed with surgery and chemotherapy twelve years ago. He had no history of previous ocular surgery or trauma and was not using any systemic medications, including corticosteroids. There was a positive family history of glaucoma, with the patient’s older sister being diagnosed at age 52, although she had not required surgery or developed significant visual impairment.

**Figure 1:**
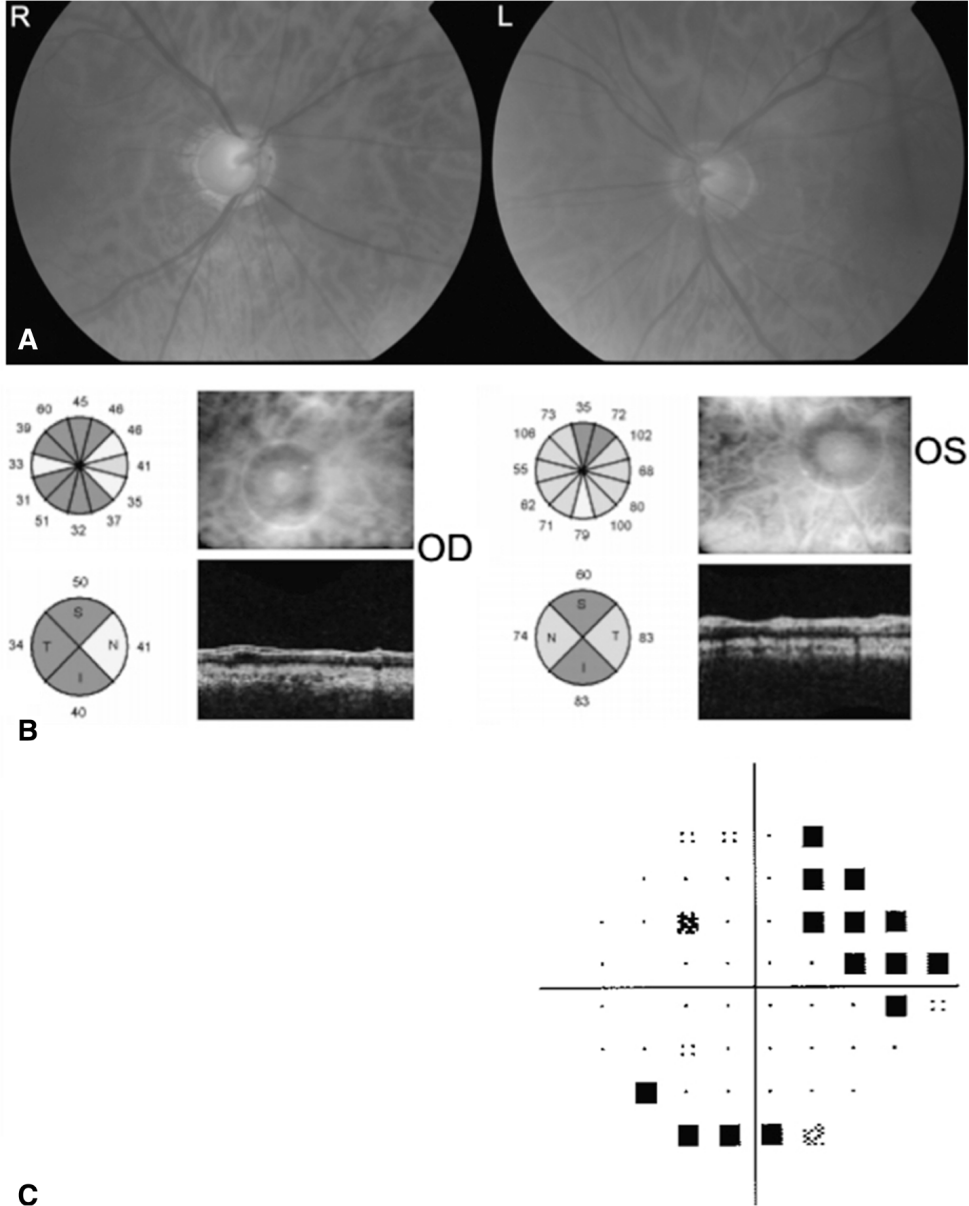
**Investigations conducted at presentation. (A)** Fundoscopic examination showing advanced glaucomatous excavation bilaterally. **(B)** Optical coherence tomography report demonstrating asymmetric RNFL thinning more pronounced in the right eye. **(C)** Left eye pattern deviation plot from standard automated perimetry.

On examination, best-corrected visual acuity was hand movements in the right eye, and 6/6 in the left. There was no injection of the conjunctivae; corneas were clear and anterior chambers deep and quiet. Both pupils were round and symmetrical in size, however there was a right relative afferent pupillary defect. Goldman applanation tonometry revealed a very high intraocular pressure (IOP) in both eyes, measuring 50 mmHg in the right and 48 mmHg in the left. Central corneal thickness was 596 μm in the right eye and 597 μm in the left. Refractive error measured only +0.50 diopter sphere in both eyes. Gonioscopy demonstrated wide-open angles bilaterally, the trabecular meshwork was heavily pigmented but there were no iris transillumination defects. On dilated fundal examination there was evidence of advanced glaucomatous excavation of both optic nerve heads, particularly severe in the right eye, where there was almost complete loss of the neuroretinal rim (Figure 1A). Optical coherence tomography (OCT) showed significant thinning of the retinal nerve fiber layer (RNFL) in both eyes, again worse in the right, with average RNFL thicknesses of 41.28 μm in the right eye and 75.11 μm in the left (Figure 1B). Standard automated perimetry was not possible in the right eye due to the poor visual acuity, but in the left eye was reliable with a superonasal defect and mean deviation of -6.54 dB (Figure 1C).

A diagnosis of advanced primary open angle glaucoma was made and the patient was commenced on topical latanoprost and brinzolamide to both eyes. After 24 hours IOPs had decreased to 28 mmHg (right eye) and 21 mmHg (left eye). Visual acuity had improved to 6/60 in the right eye, and 6/5 in the left eye. Although there had been a good percentage reduction in IOP with medication, due to the advanced glaucomatous damage a lower target pressure was required and the decision was made to proceed with Mitomycin C-augmented trabeculectomy to the right eye. Surgery was performed four weeks after presentation. During surgery the eye was treated with 0.2 mg/ml Mitomycin C (Kyowa Hakko Kirin Co, Ltd., Tokyo, Japan) for 3 minutes, and 3 10-0 nylon releasable sutures were used to secure the scleral flap. There were no complications noted and at day one post-operative review, IOP in the right eye was 30 mmHg, decreasing to 10 mmHg with gentle ocular massage. The patient was prescribed topical dexamethasone 0.1% 2 hourly during daytime hours and chloramphenicol four times per day to the right eye.

**Figure 2:**
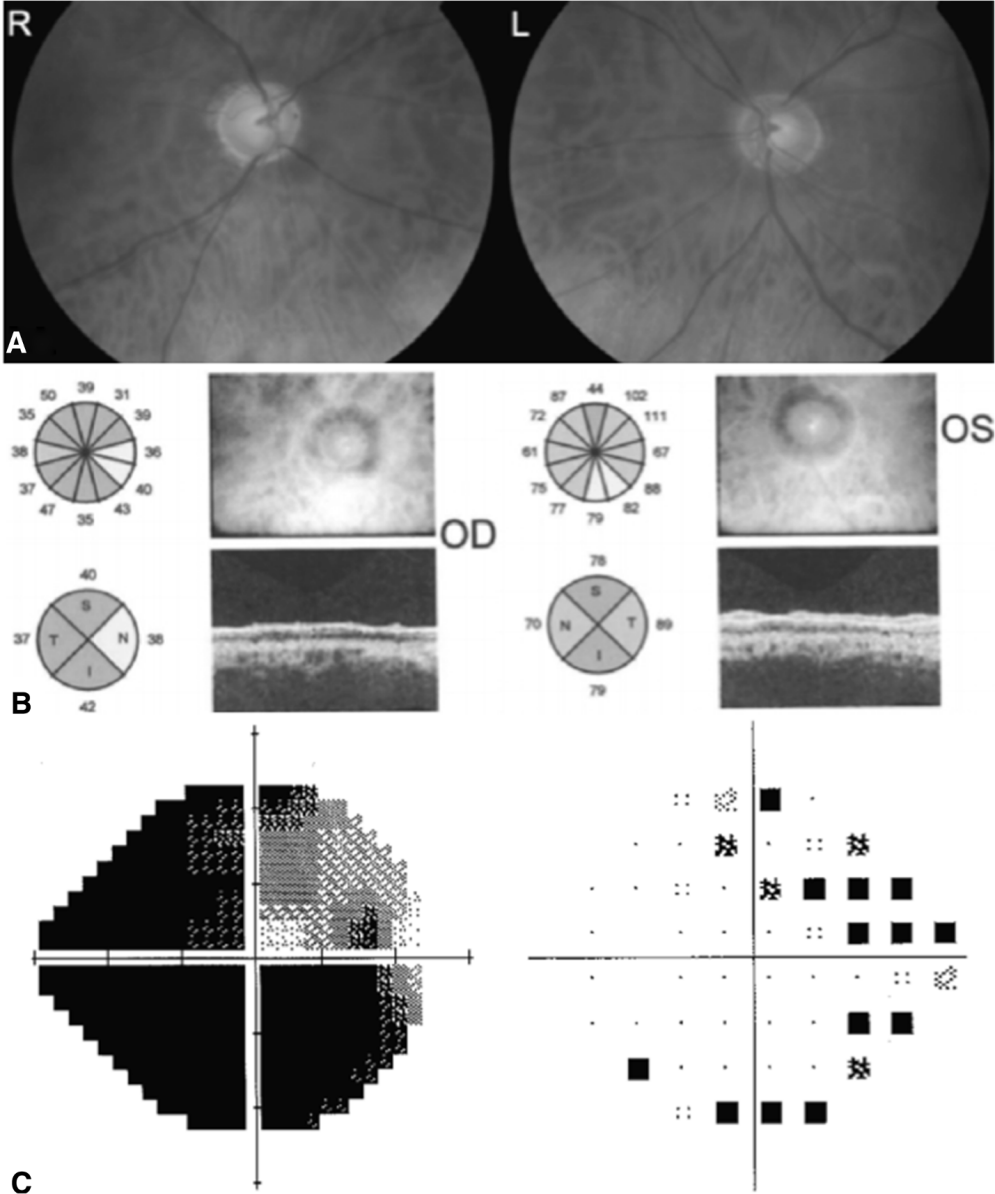
**Investigations conducted at 6 months follow-up (post-trabeculectomy). (A)** Fundoscopic examination. **(B)** Optical coherence tomography. **(C)** Right eye grey scale and left eye pattern deviation plots from standard automated perimetry.

Over the next 2 to 3 weeks IOP was gradually lowered through loosening of the releasable sutures and at the 4 week postoperative review was 9 mmHg. Surprisingly, the patient reported a gradual improvement in vision in the right eye since surgery, and at the 4 week visit the best-corrected visual acuity had improved to 6/18. The reduction in IOP and improvement in visual acuity has subsequently been maintained over six months of follow-up. During this period, the patient also reported a subjective amelioration of vision. Fundal photographs, OCT and standard automated perimetry were repeated six months following surgery and are shown in Figure 2. Although there was an improvement in vision, structural parameters in the right eye appeared unchanged with no change in neuroretinal rim appearance and average RNFL thickness in the right eye measuring 39.18 μm at most recent follow up.

## Discussion

The patient described in this case had severe glaucomatous damage at presentation, evident from the marked loss of neural tissue seen on optic disc examination, the presence of extreme thinning of RNFL on OCT, and the severe functional deficit, with a visual acuity of only hand movements and a relative afferent pupillary defect. Such severe disease at presentation conveys a poor prognosis and requires a low target pressure to minimize the risk of visual loss. Surgery was offered with the sole aim of preventing loss of remaining vision and with no expectation for visual recovery. Surprisingly, lowering of IOP resulted in a dramatic improvement in visual function.

Improvements in structural measurements are widely appreciated to occur following successful reduction in intraocular pressure with glaucoma surgery. For example, Kotecha and colleagues have shown using confocal scanning laser ophthalmoscopy that reversal in disc cupping can occur following trabeculectomy [10]. In a series of 22 eyes from 20 patients evaluated using spectral domain OCT, Russo and colleagues have demonstrated significant decreases in cup depth following trabeculectomy at both 1 week and 1 month postoperatively [11]. Increases in RNFL thickness measurements are less widely reported. However, in a small series of 38 eyes of 31 patients with glaucoma, Aydin and colleagues found a significant increase in circumpapillary RNFL thickness following glaucoma surgery. 31 of 38 eyes had an increase in RNFL thickness at 6 to 12 months following surgery, with a mean increase of 12.6 μm [12]. In fact there is a considerable body of evidence demonstrating reversal of structural glaucomatous damage following pressure-lowering surgical interventions, with apparent reversal of structural changes especially common in younger patients with congenital, infantile and juvenile-onset glaucoma [13–17].

In contrast to the improvements observed in structural measurements, evidence for functional improvement following glaucoma surgery is scarce. Leung and colleagues described a single case of a patient with juvenile open-angle glaucoma who recovered from an inferotemporal visual field defect following trabeculectomy [18]. However, due to the variability inherent in visual field testing detecting genuine improvement may be challenging.

Clinical interventions in glaucoma are generally judged on their capacity to reduce the incidence of progression of visual field endpoints, however, few studies have investigated whether improvements in visual function might occur. One exception was the Otago Glaucoma Surgery Outcome Study, which was a prospective case series including 841 eyes of 607 patients with primary open or closed angle glaucoma. Patients were treated with trabeculectomy and followed for an average of 7.5 years. Visual acuity was tested at each visit and categorised as ≥6/9, <6/9 but >6/120, or ≥6/120, with improvement in visual acuity defined by an improvement in class. The results demonstrated 151 of 841 eyes (18%) had an improvement in vision following trabeculectomy [19]. However, a limitation of this study was that 23% of eyes with improvement in vision underwent concurrent cataract extraction. In future surgical glaucoma studies it would be interesting to determine the true incidence of visual improvement.

It is important to consider the possible mechanism of visual improvement in our patient. Although the defining histological feature of glaucoma is loss of retinal ganglion cells and their axons, the exact mechanism of retinal ganglion cell death is not known. Retinal ganglion cell death is believed to be biphasic; with a primary insult initiating damage that provokes a cascade of events, in turn creating a noxious environment that envelops retinal ganglion cells, resulting in secondary cell degeneration [20]. Increased IOP and vascular deregulation may contribute to the primary insult, obstructing axoplasmic flow and altering microcirculation in the optic nerve. The secondary cascade is likely to involve excitotoxic damage from the accumulation of glutamate, increased intracellular calcium and resultant retinal ganglion cell apoptosis [21].

Once apoptosis has occurred it is difficult to conceive how visual function might improve, however, Swanson and colleagues have proposed that retinal ganglion cells might undergo a period of reversible dysfunction preceding apoptosis [22]. Evidence for this theory largely comes from primate studies of experimental glaucoma. In one study involving rhesus monkeys with unilateral laser-induced experimental glaucoma, Harwerth and colleagues found reductions in visual field sensitivity could be present without apparent retinal ganglion cell loss on histology [23, 24]. Marx and colleagues examined flash and pattern electroretinograms (PERG) in experimental glaucoma [25, 26]. The results showed that 50% reductions in PERG amplitude could occur in the absence of observable glaucomatous optic disc changes. A limitation of these studies is that they utilized animal models of experimental glaucoma, in which glaucoma was rapidly induced. However, similar findings have been reported in humans. For example, Ventura and colleagues conducted a study of 84 patients with suspected glaucoma and found a disproportionate reduction in PERG amplitude compared to RNFL thickness, supporting the concept that retinal ganglion cell dysfunction might precede permanent structural and functional changes [27].

The possibility of reversible RGC dysfunction has driven interest in the concepts of neuroprotection, neuroregeneration and neuroenhancement. Neuroprotection may allow preservation of retinal ganglion cells by halting the secondary cascade of glaucoma pathogenesis [28–30]. Neuroregeneration is the process of promoting the rebuilding of optic nerve axons and neuroenhancement is treatment to provide short-term improvements in function of surviving retinal ganglion cells.

The patient described in the present case presented with a visual acuity of hand movements, yet recovered 6/18 vision following trabeculectomy. In this case surgical reduction in IOP is likely to have had a neuroprotective effect, increasing the chance of preserving remaining retinal ganglion cells. However, the improvement in visual function is likely to have been due to a neuroenhancing effect of IOP reduction. Although the mechanism of improved vision cannot be proved, it is probable the reduction in IOP from the very high preoperative levels may have improved retinal ganglion cells function through restoration of axoplasmic flow and improved microcirculation to the optic nerve. Contributing factors may have included the patient’s relatively young age, high IOP and short duration of symptoms.

It is also important to acknowledge that the patient may have had an improvement in vision without surgery, and had already experienced some improvement in vision with IOP reduction with medical treatment. Unfortunately, due to the severe glaucomatous in the right eye, the visual prognosis remains poor.

## Conclusion

In conclusion, although glaucomatous damage is deemed irreversible, our patient experienced a significant improvement in vision following successful reduction in IOP. This patient’s experience is a single case, yet it contributes to the evidence that filtration surgery may lead to functional as well as structural improvement in some patients. This may be an important consideration for future clinical studies, which tend not examine possible improvements in vision with glaucoma surgery. For example, although the Advanced Glaucoma Intervention Study (AGIS) provided evidence that trabeculectomy is an effective procedure for lowering IOP and reducing the risk of visual field progression in advanced glaucoma, the study did not report whether there was improvement in vision in any patients following surgery [31]. This case also supports the recommendation that early surgery should be considered in patients presenting with advanced glaucomatous damage.

## Consent

Written informed consent was obtained from the patient for publication of this case report and any accompanying images. A copy of the written consent is available for review by the Editor-in-Chief of this journal.
